# Lifestyle and Dietary Patterns during the COVID-19 Lockdown in Bahrain: A Web-Based Cross-Sectional Study

**DOI:** 10.3390/nu15112543

**Published:** 2023-05-30

**Authors:** Jamil Ahmed, Aseel AlSaleh, Amer J. Almarabheh, Leila Cheikh Ismail, Aysha A. M. Almuqahwi, Hussa W. A. AlOmer, Ibrahim M. AlAlhareth, Sabiha J. M. Albader, Saad S. Alhajeri, Ayesha S. Al Dhaheri

**Affiliations:** 1 Department of Family and Community Medicine, College of Medicine and Medical Sciences, Arabian Gulf University, Manama P.O. Box 26671, Bahrain; jamilmga@agu.edu.bh (J.A.); amerjka@agu.edu.bh (A.J.A.); 2 Department of Clinical Nutrition and Dietetics, College of Health Sciences, University of Sharjah, Sharjah 27272, United Arab Emirates; lcheikhismail@sharjah.ac.ae; 3 Research Institute of Medical and Health Sciences (RIMHS), University of Sharjah, Sharjah 27272, United Arab Emirates; 4 Nuffield Department of Women’s & Reproductive Health, University of Oxford, Oxford OX1 2JD, UK; 5 Department of Nutrition and Health, College of Medicine and Medical Sciences, Arabian Gulf University, Manama P.O. Box 26671, Bahrain; ayshaa308@gmail.com (A.A.M.A.); hussa-alomar@hotmail.com (H.W.A.A.); arma-1416@hotmail.com (I.M.A.); hanenalbader@hotmail.com (S.J.M.A.); dr.alhajeri.saad@gmail.com (S.S.A.); 6 Department of Nutrition and Health, College of Medicine and Health Sciences, United Arab Emirates University, Al Ain 15551, United Arab Emirates; ayesha_aldhaheri@uaeu.ac.ae

**Keywords:** lifestyle, COVID-19 pandemic, dietary patterns, physical activity, sleep patterns

## Abstract

Lifestyle factors such as dietary habits, perceived weight, sleep, and physical activity worsened during the COVID-19 pandemic. Through this study, we aimed to understand the impact of COVID-19 on these lifestyle factors in Bahrain. A cross-sectional study was conducted among 1005 adult Bahrainis. Data were collected online using a structured and validated questionnaire for the assessment of eating habits, physical activity, and lifestyle during the COVID-19 pandemic. Participants were snowballed through those who agreed to answer the online questionnaire. There was a higher consumption of fast food, and a higher dependence on takeaways during the COVID-19 pandemic. About 63.5% of the participants consumed >4 meals per day, compared to 36.5% before the COVID-19. About 30% reported consumption of sugar sweetened beverages from two to three times a day. Weight loss was predominantly observed in persons exercising 1–3 times a week. The consumption of sugar-sweetened beverages was also high, with about 19% reporting drinking sweet beverages once every day, 10.6% from two to three times a day, and 40.4% from one to four times a week. A higher proportion of the participants reported poor sleep quality during the pandemic (31.2%) compared to before (12.2%), and 39.7% of the participants reported feeling lazy. Screen time use also doubled during the pandemic, with participants spending more than five hours per day on screens for entertainment, which went from 22.4% before to 51.9% during the pandemic. The lifestyle and dietary habits changed drastically for our study participants during the pandemic. The increased reliance on processed fast food instead of healthier options is a challenge to be tackled in any future pandemic situation. Future research needs to focus on strategies to promote healthier lifestyle modifications during situations such as the COVID-19 pandemic.

## 1. Introduction

The Gulf Cooperation Council (GCC) populations have one of the highest burdens of overweight and obesity in the world. About 70% of the GCC population is currently overweight, and from 29% to 37% are obese [1]. As a result, the mean Body Mass Index (BMI) has increased in the region by 1.29 kg/m² between 1980 and 2008 [1]. Non-communicable diseases, mainly Diabetes Mellitus and cardiovascular diseases, have increased so rapidly that the GCC region has the highest rate of Diabetes Mellitus in the world [1]. In every five GCC nationals one currently lives with Diabetes Mellitus, and much of this burden of obesity in the region is attributed to a culture of dietary consumption that favors weight gain [1]. For instance, only about half of the adults in Bahrain consume fruit and vegetables on a daily basis [2]. Knowledge about calories in food is low in the region [3]. This is complicated by peer pressure [4], advertisements of fast food [5], large meal consumption, and frequent eating of sweets, which all contribute to eating unhealthy food [6]. Availability and consumption of fast foods has particularly encouraged this dietary consumption pattern comprising of food high in calories but low in nutritional quality. As a consequence, about 80% of the regional population consumes fast food at least once a week [7]. The high burden of obesity resulting mainly from unhealthy dietary habits is also compounded by a lack of physical activity in the region where the populations are least active in the world [8]. For instance, about 70% of the Saudi Arabian population does not perform the recommended physical activity [1]. This high burden of risk factors of non-communicable diseases and metabolic syndrome is considered a risk factor for severe COVID-19 [9].

There is adequate evidence that suggests that pandemics may accelerate weight gain and exacerbate risk factors associated with non-communicable diseases [10]. Self-reported weight gain has also been reported from other population groups during the COVID-19 pandemic [11]. A longitudinal study in Italy reported that among obese children and adolescents, eating, physical activity, and sleep behaviors changed negatively, which could promote weight gain during the national lockdown [10]. Risk factors for obesity such as lack of sleep, decreased physical activity, eating under stress, and snacking after dinner have also been reported in adults during self-lockdowns implemented during the COVID-19 pandemic [12]. Another study from Italy showed that, after a month of lockdown, obese patients reported a weight gain of 1.5 kg. This study showed that risk factors such as a lower education level, anxiety or depression, and a lack of attention to healthier food choices were significantly associated with weight gain leading to an abnormal BMI [13]. Another study, also from Italy, in which 3533 individuals were surveyed, reported that 48.6% of the population believed that they had gained weight during the pandemic (5). Much of this weight gain could be because of changes in dietary patterns during the pandemic [14]. 

Poor diet is suggested to contribute to higher mortality from COVID-19 infection [15,16]. Following a balanced dietary routine, comprising a healthy and complete diet, and vitamin and mineral supplementation, is crucial to preventing and addressing comorbidities associated with COVID-19 and related complications [14,17,18]. This is because poor nutrition contributes to worsening or improper control of hypertension, Diabetes Mellitus, and obesity, impairing immunity, and increasing vulnerability to the complications from COVID-19 [18]. Furthermore, spending more time at home, for example, while working or receiving education from home, increased people’s exposure to processed and ultra-processed foods, and food with high glycemic index [19,20]. A study from Poland showed a 34% increase in food consumption at home [21]. A population-based, cross-sectional study that assessed eating habits and lifestyle behaviors among residences of the UAE via an online survey reported that the COVID-19 pandemic and the subsequent lockdown resulted in weight gain in about one-third of the study population. An increase in the number of meals consumed per day and a reduction in the proportion of meals skipped during the day, especially breakfast, have also been reported [22]. A study from Kuwait showed a fourfold increase, for instance, in late-night snacking [23]. 

Although the stress of lockdowns during the pandemic has led to unhealthy dietary habits, in other cultures such as in France, people have opted for healthier and more sustainable food choices [24]. Healthier food choices, including a higher intake of fruits, vegetables, or legumes and a lower intake of processed red meat, alcohol, fried foods, or pastries compared to the usual habits, have also been reported in the Spanish COVIDiet Study, in which 7514 people answered the survey [25]. Similarly, another study reported that during the pandemic, overall scores for healthy eating increased due to a decline in eating out in restaurants, and increased use of home cooked food [26]. Much of this realization and awareness about healthier foods has arisen from people’s perceptions that maintaining good health is akin to better immunity and resistance against COVID-19 [27].

Stress as a result of the COVID-19 pandemic has also been one of the key factors determining altered dietary patterns and habits [28]. Anxiety has also increased, especially among obese individuals [26]. Emotional eating was also observed among women during the COVID-19 pandemic and was strongly associated with gestational weight gain during the pandemic [29]. Social distancing during the COVID-19 quarantine was reported to be associated with emotional eating and weight gain (2 kg) among 765 patients who visited the bariatric clinic in Iraq [30]. Poor-quality sleep is also associated with weight gain and can impact mental health [28,31,32]. A study from the region showed that sleep was affected during the pandemic [23]. In China, stressful eating was shown to be associated with unhealthy eating [33]. Not only that, but lockdowns have also led to higher use of tobacco and alcohol, which also impact mental wellbeing [34]. 

During the current COVID-19 pandemic, several studies have investigated the increased unstructured time spent indoors and risk factors of obesity [35]. Physical activity rates have also been reported to have declined during the pandemic, leading to a negative impact on mental and physical wellbeing [32]. Conversely, a deterioration of mental health because of the pandemic has also been implicated in a reduction in physical activity levels [36]. Sedentary behavior because of confinement to homes because of lockdowns and industry shifting work from offices to homes has increased, while time spent in physical activity has declined [32,37]. This is because restrictions during lockdowns to contain the spread of the epidemic have limited people’s access to places where they previously performed exercises [38]. Almost half of the participants in a cross-sectional study from Poland were less active than before the COVID-19 pandemic [21]. However, a study from Kuwait showed only a slight change in physical activity from before to after the pandemic; this was probably because the population was already less physically active before the pandemic [23]. While a lack of physical activity itself is associated with obesity, a recent study from Brazil also showed a higher incidence of unhealthy diets associated with sedentary behavior [39]. Physical activity is also associated with better control over stress and unhealthy eating habits [40,41]. The present study was conducted to measure the impact of the COVID-19 pandemic on dietary practices, physical activity, and lifestyle patterns in Bahrain. The findings from this study may guide policymakers and communities in dealing with the impact of a pandemic on peoples’ wellbeing. This study aimed to determine the effect of COVID-19 pandemic and resulting lockdowns on reported dietary patterns and lifestyle (sleep patterns, stress, and physical activity) among adults, ≥18 years, in Bahrain. 

## 2. Methods 

Research design and methodology: this cross-sectional study was conducted among adults, ≥18-year-old, Bahrainis. The sample consisted of adults volunteering to participate from Bahrain. The participants answered the questionnaire from March to September 2021. The point of time “before” was defined as the time period earlier than 30 January 2020 when WHO declared it a Public Health Emergency of International Concern (PHEIC). 

Data collection: data were collected online by using a set of validated questionnaires for the assessment of eating habits and lifestyle during the COVID-19 pandemic. The questionnaire had previously been used to evaluate changing patterns of lifestyle and diet during the COVID-19 pandemic. These questionnaires assessed participants’ dietary habits, physical activity, and stress before and during COVID-19. The dietary assessment questionnaire is a validated tool used previously in a published study [22]. The tool was used in collaboration with these researchers. For the physical activity and change in the physical activity during COVID-19 pandemic, the International Physical Activity Questionnaire Short Form (IPAQ-SF) was used. The Copenhagen Psychosocial Questionnaire (COPSOQ-II) was used for the assessment of sleep disturbance. The questionnaires were administered in Arabic. The questionnaire was divided into several parts, which comprised questions on socio-demographic characteristics, sources of information related to lifestyle and eating patterns, evaluation of eating habits, shopping habits, physical activity, stress and irritability, and sleep patterns.

The total number of individuals who answered the questionnaires was determined by the end of the data collection period, which was about seven months from the date of the start of the data collection. A sample of about 1000 individuals was expected to conduct advanced statistical analyses. Participants were snowballed through those who agreed to answer the online questionnaire. Participants were invited through a link to the questionnaire available on Google survey sent to them through social media, including WhatsApp, Facebook, LinkedIn, and by emails. 

Inclusion and Exclusion Criteria: adult males and females, ≥18 years, were eligible to participate in the study. Pregnant women and persons with severe illnesses were exempted from answering the questionnaire. Males and females were included equally in the study. Participants with special needs were also searched so that we could also include them in our study. 

Statistical analysis: the data were first retrieved from the Google Forms as Excel sheet and then exported to the Statistical Package for Social Sciences version 28 for analysis. Next, the data were cleaned and organized, and categories were assigned value labels according to the English version of the questionnaire. Initially, frequency tables were created to make decisions about analyzing outcomes and independent variables. The Pearson’s chi-square (χ^2^) test of significance was used to assess the statistical differences between outcome and independent variables. Minitab (version 14) software was used to investigate the significant difference between two proportions for the categorical variables before and during the COVID-19. Thus, a univariate analysis was performed to determine statistically significant differences between outcomes such as eating habits, the frequency of consumption of foods during the COVID-19 pandemic, sleep patterns before and during the COVID-19 pandemic, and any change in physical activity levels and perceived weight during the pandemic. These variables were cross-tabulated according to their prevalence before and during the COVID-19 pandemic. A 5% level of significance was considered to evaluate the differences across the variables. 

Ethical consideration: the consent form was placed at the start of the Google Form. The purpose of the study was mentioned at the beginning of the form, and it was clarified in the form that participation in the study would be voluntary, and that the identity of the participants would be confidential and anonymous. The contact details of the ethical review committee were provided in case the participants, or their institutions had any questions about the ethical conduct of the study. The data from this study were stored in a password-protected computer accessible only to the investigators.

## 3. Results

### 3.1. Demographic Characteristics

The questionnaire was completed by 1005 participants. A vast majority, 84.2%, were female. All participants answered the Arabic version of the questionnaire. Most of the participants (77.4%) were from 18 to 25 years old, whereas less than 1% of those over 55 years old answered the questionnaire. Likewise, the majority (80.8%) of the participants were not married; therefore, about 85.7% said that they did not have any children. Half (50.3%) of the participants said that they had graduated from a university, about 38% had completed high school, and about 5% had achieved higher education. Most of the participants (68.1) were self-employed (Table 1).

### 3.2. Sources of Information

Participants were asked about their sources of health and nutrition information during the COVID-19 pandemic. We found that websites and social media were the major sources of health and nutrition-related information for our participants. Specifically, websites and social media were the source for health-related information for 80.7% and nutrition-related information for about three-quarters (76.5%) of the sample, whereas 69.5% and 50.7% relied, respectively, on local and international health authorities for health- and nutrition-related information (Table 2).

### 3.3. Eating Habits

Table 3 presents a comparison of the eating habits of the participants before and during the pandemic. Although consumption of homemade, frozen, ready-to-eat meals, and fast food (takeout and delivery) increased slightly during the pandemic, the differences were not statistically significant (*p* > 0.05). Consumption in restaurants other than fast food, however, decreased from 8% before to 5.5% during the pandemic (*p* = 0.026). There were about 12% of participants who reported that they ate more than four meals a day during the pandemic, which was 5% higher than before the pandemic (*p* ≤ 0.001). Similarly, about half (49.7%) of the participants reported not skipping their meals during the pandemic, compared to 44.4% before (*p* = 0.018). When queried about their water consumption, the participants reported higher consumption of water during the pandemic than before; for instance, 19% reported drinking eight or more cups of water during the pandemic, compared to 15.3% before the pandemic (*p* = 0.028).

### 3.4. The Frequency of Consumption for Specific Food

Table 4 gives details of the frequency of consumption of particular foods during the COVID-19 pandemic in Bahrain. We found that only 2.8% of the participants followed the recommended guidelines of eating four servings of fruit and vegetables once a day. Furthermore, 9% and 6.6% of respondents, respectively, said they never ate fruits or vegetables. However, meat consumption was more than adequate, with 37.1% reporting eating meat once daily, 31.5% from two to three times a day, and 26.3% from one to four times a week. Like meat, the consumption of carbohydrate-rich foods such as bread, pasta, and rice was also high among the sampled population, with 32.3% consuming them daily, 36% from two to three times daily, and 24% from one to four times a week. A similar pattern of consumption was observed for sweets and deserts, with about a third (32.8%) of the participants reporting consuming this type of food once daily, but about half of the participants consumed it at least twice or more than four times a day. Consumption of sugar-sweetened beverages was high in our study. About 19% reported drinking sweet drinks once daily, 10.6% from two to three times a day, and 40.4% from one to four times a week.

### 3.5. Sleep

Table 5 shows a significant increase in proportions of participants who reported sleeping more than nine hours per night from 9.1% before to 16.3% during the pandemic (*p* < 0.001). However, a higher proportion of the participants reported poor sleep quality during the pandemic (31.2%) compared to before (12.2%) (*p* < 0.001). Consequently, 39.7% of the surveyed participants reported feeling lazy and less energized during the pandemic, compared to only 7.5% before the pandemic (*p* < 0.001).

### 3.6. Physical Activity

A decrease in physical activity during the COVID-19 pandemic was more frequently reported among participants who had a more active lifestyle before the pandemic (*p* < 0.001) for performing household chores 1–3 times per week. The results indicated a significant increase in the percentage of participants who performed household chores every day, from 27.2% before the pandemic to 31.9% during the pandemic (*p* < 0.019). Regarding the time spent on daily work or study, the results showed that 66.3% of the participants spent more than five hours per day for study or work on the computer during the pandemic, compared to 14.9% before (*p* < 0.001). Similarly, the percentage of participants spending more than five hours per day on screens for entertainment increased from 22.4% before to 51.9% during the pandemic (*p* < 0.001) (Table 6).

More participants who never performed any exercise gained weight (52%) than participants who lost or maintained during the pandemic. While the participants who exercised from one to three times per week had the highest rates of perceived weight gain (45.7%), the participants who exercised from four to five times a week or daily had a weight gain, respectively, of 27.4% and 35.5%. Those performing exercise daily also reported the highest rate of weight loss (84.4%). These results were tested by the chi-square test, which indicated that these differences of exercise during the pandemic and weight change were statistically significant (χ^2^ = 52.578, *df* = 6, *p* < 0.001) (Table 7).

## 4. Discussion 

This cross-sectional study examined the lifestyle-related behavior and eating patterns of adult Bahrainis during the COVID-19 pandemic. Overall, we found significant differences in the lifestyle and eating patterns of the Bahraini population during the pandemic in comparison to before. The results showed that the participants’ perceived weight slightly increased by almost 1.58 kg during the pandemic compared to before. However, because many participants reported that they were increasingly involved in household chores and other types of physical activities, we found that 84.4% of those who lost weight performed exercise every day. In our study, consumption of meals increased during the pandemic, as 12% of participants reported that they ate more than four meals a day during the pandemic, which was 5% higher than before the pandemic. Additionally, 49.7% of the participants reported not skipping their meals during the pandemic, compared to 44.4% before. On the other hand, the consumption of healthy foods showed a decline during the pandemic. For example, the study found that only 2.8% of the participants followed the recommended guidelines of eating four servings of fruit and vegetables once a day. Participants also reported consuming sweets and desserts more frequently during the pandemic compared to before, with 32.8% of the participants reporting consuming sweets and desserts once daily, with about half of the participants consuming them at least twice or more than four times a day. In addition to this, the consumption of sugar-sweetened beverages was also high, with about 19% reporting drinking sweet beverages once every day, 10.6% from two to three times a day, and 40.4% from one to four times a week. Participants’ sleep was also reported to be affected during the pandemic; a higher proportion of the participants reported poor sleep quality during the pandemic (31.2%) compared to before (12.2%). Consequently, 39.7% of the surveyed participants reported feeling lazy and less energized during the pandemic, compared to only 7.5% before the pandemic. Screen time use also seemed to have more than doubled during the pandemic, with participants spending more than five hours per day on screens for entertainment, which went from 22.4% before to 51.9% during the pandemic.

This study showed some perceived weight gain in the adult population in Bahrain during the pandemic. Although this result is based on participants’ reporting their weight themselves and body mass index could not be calculated because information about height was not obtained due to concerns of inaccuracy, the mild weight gain reported by the participants is still significant and corroborates a widely reported trend of weight gain during the pandemic. Our study’s findings about weight gain during lockdowns and stay-at-home orders are consistent with similar findings reported from most other regions of the world, indicating a strong association between staying at home and weight gain. A large population-based study from the USA reported a weight gain of about 7 kg in the adult population during the pandemic, with people of low socioeconomic groups affected disproportionately [42]. Studies from the European region also reported an increase in weight in the general population; for instance, an online survey found that 58.5% of the sample reported weight gain during the pandemic [43]. Similarly, adults in the USA also gained 0.6 kg on average [44]. Studies have also shown that obese [44,45], anxious, depressed individuals, and females [46] gained more weight than their counterparts [47]. In Malaysia, 45.5% of the participants reported gaining about 1.61 kg of weight a month following the lockdowns [48].

Our findings of consuming a greater number of meals and half of the participants not skipping their meals during the lockdowns of the pandemic are also consistent with findings from other similar studies. Our results also concur with a previous similar study from the region, which also showed a high consumption of unhealthy foods, where 46% and 37% of the participants consumed sweets and desserts and a salty snack at least once per day [22]. A study from Jordan showed a higher prevalence of snacking between meals. This study also showed that 62% of their participants were aware of their health and consumed healthy foods [49]. In another study, more than half of the participants reported increased food intake, frequent snacking, and ordering food online during the lockdown [48]. The consumption of homemade and takeaway fast food and desserts was also high during the lockdowns. For instance, a study showed that 54% of the individuals ate homemade desserts, sweet drinks and meat were two of the most frequently consumed food items in overweight or obese individuals [48]. Not only did the number of meals, snacks, and unhealthy food consumption increase, but the serving size also increased, leading to a higher risk of weight gain [43]. Our finding that only 2.8% of the participants followed the recommended guidelines of consuming four servings of fruit and vegetables a day, or a high consumption of carbohydrate-rich foods such as bread, pasta, and rice, or sweets, deserts, and sweet drinks, shows a pattern of a predominantly unhealthy diet in the study population. The findings are consistent with those of other recent research identifying similar unhealthy dietary choices during the pandemic [22]. 

Our study reported that 31.2% of the participants had poor-quality sleep during the lockdown periods, compared to 12.2% before, with about 39.7% reporting being lazy. Our study reported a higher difference in sleep quality than a previous study carried out in the region, which showed a poor sleep quality of 28% during the pandemic compared to 17.3% before the pandemic [22]. Another study from the region reported lower sleep efficiency, another measure of sleep quality, of 25.5% in females and 13.3% in males [50]. Sleep quality has been reported to have been affected by the pandemic, probably because of the pandemic-related challenges impacting people’s lifestyles and mental wellbeing [51] and because of their fears and the spread of misinformation about the pandemic [52]. It is also important to note that poor sleep quality has been attributed to stressful factors of the COVID-19 pandemic, including lockdowns and news about the pandemic [53] and that this stress could be higher in certain groups of populations than others. For instance, the sleep disturbance could be worse in the survivors of COVID-19, as a study showed that about one-third of them suffered from sleep disorders [54]. Our findings are consistent with a study from Brazil, which reported a prevalence of impaired sleep quality in lockdowns of 42%, attributing this to increased alcohol consumption, poor-quality diet, and lying down on the bed while working from home during lockdown [55]. Another study that assessed sleep quality in healthcare students showed that three-fourths of the students suffered from poor sleep quality [56]. 

We reported that exposure to screen time of more than 5 h doubled from 22.4% before to 51.9% during the pandemic. This is more than reported by a similar regional study, which showed that 36.2% of the participants spent over five hours on screens daily [22]. A study from Jordan showed increased sitting and screen time, while 74% of the participants felt more stressed and anxious [49]. Higher screen time has also been reported to be associated with negative consequences such as weight gain. For instance, a study reported that exposure to screens for 3–6 h a day was associated with higher weight gain compared to those who were rarely exposed to screens [46]. Exposure to prolonged screen time was reported not only in adults but also in adolescents. A Swedish cohort study that followed adolescents before the pandemic and assessed the participants during the pandemic found that adolescents had a mean screen time of 307 ± 101 min (>5 h per day) during the weekends [57]. A study from the UAE found even higher screen time in adolescents, at 420 min per day, with more time spent on screens during the weekends compared to weekdays [58]. 

We believe that working from home and distant education by online methods were associated with increased time spent at home, leading to greater consumption of and snacking on food during the pandemic, particularly when lockdowns were in place or when the fear of contracting the virus was high in the communities. This shift in work and education from the workplace to the home was unprecedented, lifting restrictions of space and availability such as those at workplaces, leading to an accumulation of irresistible eating and irregular eating patterns. People are probably more likely to indulge in sweets and other unhealthy foods when at home because of the ease of access to and availability of food. Another factor that may have caused increased consumption of meals among our participants could be that since the restaurants closed their dine-in options, there has been an increased use of online ordering and takeout.

## 5. Limitations

The study used a large sample of a homogenous population in Bahrain and reported the lifestyle-related factors of this population comprehensively using a validated tool for the first time, which is a strength of this study. Nevertheless, the study is not without some limitations. We acknowledge that weight measurements were not possible as the study was conducted by sending an online questionnaire, and results are based on self-reported or perceived weight. Furthermore, despite the fact that most people possess a weight scale at home, some people may not be aware of their actual weight and may have reported a lower or higher weight, raising questions of inaccuracy in the reporting of weight. However, since most studies also report weight gain during the pandemic, our findings are comparable. The online nature of the study led to a higher representation of younger people compared to older people because the latter use the internet more. Because of this, the findings may not be generalizable to older people, whose representation in the sample is not adequate. Moreover, as we used a self-report questionnaire, there is the possibility of a recall bias, which might have affected the results; however, a careful comparison of the study’s findings with international literature shows that the results are comparable.

## 6. Conclusions

Although participants reported increased involvement in physical activities and household chores, overall, factors such as eating higher number of meals, poor-quality sleep, and high exposure to screens during the pandemic suggest that lifestyle was negatively affected during the epidemic. The impact of the lockdowns and fears about the COVID-19-related news may have contributed to the negative impact on the psychological wellbeing of the communities in Bahrain, which could have led to a higher prevalence of these negative lifestyle factors. This warrants further studies to assess the accurate burden of the negative impact of the pandemic in other similar communities.

## Figures and Tables

**Table 1:** Demographic characteristics of study participants (*n* = 1005).

Characteristics	*n*	%
Gender		
Female	846	84.2
Male	159	15.8
Age (years)		
18 to 25	778	77.4
26–35	136	13.5
36–45	62	6.2
46–55	20	2.0
More than 55	90	0.9
Marital status		
Married	185	18.4
Single	812	80.8
Divorced or widowed	8	0.8
Number of children		
None	862	85.7
1–2	75	7.5
≥3	68	6.8
Education level		
Less than high school	33	3.3
High School	380	37.8
College/Diploma	37	3.7
University Degree	505	50.3
Higher education masters/doctorate	49	4.9
Employment status		
Self-employed	684	68.1
Full-time	192	19.1
Unemployed/Retired	106	10.5
Part-time	13	1.2
Retired	10	1.0

**Table 2:** Source of health and nutrition information during COVID-19 pandemic (*n* = 1005).

Source of Information *	Health-Related Information *n* (%)	Nutrition-Related Information *n* (%)
Local and international health authorities	690 (69.5)	505 (50.7)
Websites and social media	760 (76.5)	805 (80.7)
Healthcare professionals	483 (48.6)	492 (49.3)
Television	89 (9)	68 (6.8)
Newspapers	23 (2.3)	23 (2.3)
Friends and family	418 (42.1)	453 (45.4)

* As multiple responses were allowed, the total number of responses is greater than the number of surveyed participants and the percentage of cases is displayed.

**Table 3:** Eating habits pre- and during COVID-19 pandemic (*n* = 1005).

Variables	Pre-COVID-19 *n* (%)	During COVID-19 *n* (%)	*p* Value
Most-consumed meals during the week			
Homemade	627 (62.4)	614 (61.1)	0.551
Frozen ready-to-eat meals	53 (5.3)	64 (6.4)	0.295
Fast food (take-away, delivery)	223 (22.2)	243 (24.2)	0.290
Restaurants (take-away, delivery)	80 (8.0)	55 (5.5)	0.026
Healthy food (take-away, delivery)	22 (2.2)	29 (2.9)	0.321
Number of meals per day			
1–2 meals	344 (34.2)	364 (36.2)	0.350
3–4 meals	591 (58.8)	519 (51.6)	0.001
More than 4 meals	70 (7.0)	122 (12.1)	<0.001
Eating breakfast on most days			
Yes	551 (54.8)	568 (56.5)	0.445
No	454 (45.2)	437 (43.5)	
Skipping meals			
Yes	559 (55.6)	506 (50.3)	0.018
No	446 (44.4)	499 (49.7)	
Amount of water consumed per day			
1–4 cups	580 (57.7)	476 (47.4)	<0.001
5–7 cups	271 (27.0)	338 (33.6)	0.001
8 cups and above	154 (15.3)	191 (19.0)	0.028

**Table 4:** The frequency of consumption of specific, common, and local foods during COVID-19 pandemic (*n* = 1005).

Food Items	>4 Times/Day *n* (%)	2–3 Times/Day *n* (%)	Once/Day *n* (%)	1–4 Times/Week *n* (%)	Never *n* (%)
	*n* (%)				
Fruits	28 (2.8)	100 (10)	255 (25.4)	527 (52.4)	95 (9.5)
Vegetables	28 (2.8)	174 (17.3)	325 (32.3)	409 (40.7)	69 (6.9)
Milk and milk products	30 (3)	212 (21.1)	324 (32.2)	365 (36.3)	74 (7.4)
Meat/Chicken/Fish	31 (3.1)	317 (31.5)	373 (37.1)	264 (26.3)	20 (2)
Bread/rice/pasta	61 (6.1)	361 (35.9)	325 (32.3)	241 (24)	17 (1.7)
Sweets/desserts	43 (4.3)	188 (18.7)	330 (32.8)	384 (38.2)	60 (6)
Coffee/Tea	108 (10.7)	297 (29.6)	246 (24.5)	239 (23.8)	115 (11.4)
Sweet drinks (soft drinks, canned juice, etc.)	26 (2.6)	107 (10.6)	189 (18.8)	406 (40.4)	277 (27.6)
Energy drinks	5 (0.5)	16 (1.6)	35 (3.5)	111 (11)	838 (83.4)

The participants’ self-reported weight increased by almost 1.58 kg during the pandemic compared to before. The sample mean weight before COVID-19 pandemic was 62.61 ± 17.95, and during 63.87 ± 18.56 (*p* < 0.001).

**Table 5:** Sleep pre- and during COVID-19 pandemic (*n* = 1005).

Variables	Pre-COVID-19 *n* (%)	During COVID-19 *n* (%)	*p* Value
	Number of hours of sleep at night		
Less than 7 h	407 (40.5)	385 (38.3)	0.315
7–9 h	507 (50.4)	456 (45.4)	0.023
More than 9 h	91 (9.1)	164 (16.3)	<0.001
	Sleep quality		
Very good	377 (37.5)	232 (23.1)	<0.001
Good	505 (50.2)	459 (45.7)	0.040
Poor	123 (12.2)	314 (31.2)	<0.001
	Energy level		
Energized	438 (43.6)	117 (11.6)	<0.001
Neutral	492 (49.0)	489 (48.7)	0.894
Lazy	75 (7.5)	399 (39.7)	<0.001

**Table 6:** Entertainment pre- and during COVID-19 pandemic (*n* = 1005).

Doing Household Chores	Pre-COVID-19 *n* (%)	During COVID-19 *n* (%)	*p* Value
1–3 times/week	651 (64.8)	573 (57.0)	<0.001
4–5 times/week	81 (8.1)	111 (11.0)	0.023
Every day	273 (27.2)	321 (31.9)	0.019
Time spent daily on the computer (work/study)			
Never	237 (23.6)	73 (7.3)	<0.001
1–3 h	408 (40.6)	72 (7.2)	<0.001
3–5 h	210 (20.9)	194 (19.3)	0.373
More than 5 h	150 (14.9)	666 (66.3)	<0.001
Screen time for entertainment			
Less than 30 min/day	70 (7.0)	44 (4.4)	0.012
1–2 h/day	322 (32.0)	124 (12.3)	<0.001
3–5 h/day	388 (38.6)	315 (31.3)	0.001
>5 h/day	225 (22.4)	522 (51.9)	<0.001

**Table 7:** The association between perceived weight change and exercise during COVID-19.

Physical Activity	Perceived Weight Change during COVID-19 Pandemic			*p* Value
	Lost Weight (*n* = 272)	Gained Weight (*n* = 465)	Maintained Weight (*n* = 268)	
Never	78 (17.9)	226 (52.0)	131 (30.1)	<0.001
1–3 times/week	125 (29.5)	194 (45.7)	105 (24.8)	
4–5 times/week	39 (46.4)	23 (27.4)	22 (26.2)	
Every day	30 (84.4)	22 (35.5)	10 (16.1)	
